# The Association of Periodontal Inflammation and Systemic Health Indicators: A Machine Learning Approach

**DOI:** 10.1111/jcpe.70000

**Published:** 2025-07-23

**Authors:** Yumeng Yan, Praveen Sharma, Jeanie Suvan, Francesco D'Aiuto

**Affiliations:** ^1^ Periodontology Unit, UCL Eastman Dental Institute London UK; ^2^ Periodontal Research Group, School of Dentistry, Institute of Clinical Sciences University of Birmingham Birmingham UK; ^3^ National Institute for Health Research, Birmingham Biomedical Research Centre Birmingham UK; ^4^ Birmingham Dental Hospital, Birmingham Community Healthcare NHS Foundation Trust Birmingham UK; ^5^ Oral Sciences, University of Glasgow Dental School, School of Medicine, Dentistry and Nursing, College of Medical, Veterinary and Life Sciences University of Glasgow Glasgow UK

**Keywords:** C‐reacive protein, inflammation, periodontal inflamed surface area, periodontitis, systemic diseases

## Abstract

**Aim:**

The relationship between oral and systemic inflammation has profound implications for understanding the broader health impacts of periodontitis. The aim of this study was to (a) explore the association between periodontal inflammation and markers of systemic inflammation and metabolic health, and (b) preliminarily assess periodontal status based on systemic health indicators using machine learning techniques.

**Methods:**

Data from a cross‐sectional cohort (*N* = 667) were modelled (simple/multiple linear, fractional polynomial, logistic and random forest regression) to examine the association between systemic and periodontal measures. Three classifiers—random forest (RF), support vector machine (SVM) and gradient boosting (GB)—were used using periodontal inflamed surface area (PISA) and demographic and anthropometric variables (age, gender, ethnicity, body mass index [BMI] and smoking habits) as inputs to predict systemic inflammation (defined using serum C‐reactive protein [CRP] levels). The best performing classification models (evaluated using area under the curve, AUC analyses) were validated using a second nationally representative dataset from the National Health and Nutrition Examination Surveys (NHANES) 2001–2002 and 2003–2004 combined datasets (*N* = 2288). Next, RF, SVM and GB were employed incorporating a set of systemic parameters (including serum CRP and lipid profiles) to predict the diagnosis of periodontitis. The best performing classification models were then validated using the NHANES 2009–2010 (*N* = 664) dataset.

**Results:**

A nonlinear trend of CRP levels and PISA was confirmed by fractional polynomial regression (*p* = 0.008). Further, multiple linear regression analyses (adjusted for age, gender, ethnicity, BMI and smoking habits) confirmed a statistically significant relationship between log‐transformed CRP levels and PISA (*p* < 0.0001). Logistic regression confirmed a relationship between PISA and low‐density lipoprotein (LDL) in both crude and adjusted models. Among the classification models, SVM showed the highest performance in distinguishing CRP < 2 mg/L from CRP ≥ 2 mg/L (AUC = 0.71). The SVM model was successfully replicated in the NHANES 2001–2002 and 2003–2004 waves (AUC = 0.74). Prediction of periodontitis status (case vs. control) based on systemic indicators using the SVM model achieved the best performance with a mean AUC of 0.82. This was partially confirmed after external validation using the 2009–2010 NHANES dataset (AUC of 0.72).

**Conclusion:**

This study confirmed a consistent relationship between measures of cumulative periodontal inflammation and systemic inflammation through machine learning models. A predictive model incorporating systemic health parameters helped in identifying a case with periodontitis. Both models have potential for use in primary healthcare settings, including screening programmes, as providing confirmation of the bidirectional link between periodontitis and systemic health.

## 1 Introduction

Periodontitis is a highly prevalent (46%–69.3%) disease initiated by bacterial dysbiosis in susceptible individuals (Murakami et al. 2018) and represents a global public health problem (Petersen and Baehni 2012). Indeed, it places a substantial financial burden on healthcare systems and affects the quality of life, especially for those with the most severe forms (Botelho et al. 2022).

Periodontitis is not only limited to the oral cavity but has links to adverse systemic health outcomes (Hajishengallis and Chavakis 2021). It has been reported that patients with periodontitis exhibit higher systemic inflammation compared to healthy controls (Sanz et al. 2020). Epidemiological evidence has further revealed that poor periodontal health is associated with disorders in the cardiovascular system, metabolic system, brain and kidney (Gaur and Agnihotri 2015; Khader et al. 2004; Nibali et al. 2013; Parsegian et al. 2022). Common risk factors for both cardiovascular and metabolic diseases—including age, gender, ethnicity, environmental exposures (e.g., smoking), obesity and genetic predispositions (Kaur 2014; Mozaffarian et al. 2008)—also contribute to the development of periodontitis (Genco and Borgnakke 2013). Additionally, bidirectional relationships have been reported between periodontitis and specific systemic disorders including diabetes (Fentoglu and Bozkurt 2008; Nagpal et al. 2015). This suggests the potential for predicting systemic inflammation from periodontal status, and vice versa.

C‐reactive protein (CRP) is the most widely used and stable biomarker of systemic inflammation (Pepys and Hirschfield 2003). CRP has been implicated in the development and instability of atherosclerotic plaques, which are the key drivers of coronary artery disease, stroke and peripheral artery disease (Carra et al. 2023). To quantify the association between periodontal inflammatory burden and systemic inflammation indicators is an area of ongoing investigation. Quantifying the periodontal inflammatory burden could help in defining its link with systemic health outcomes (Van Dyke et al. 2018). Periodontal inflamed surface area (PISA) represents the total surface area of the inflamed gingival epithelium (Nesse et al. 2008). PISA incorporates measures of bleeding on probing (BoP) and probing pocket depth (PPD) or clinical attachment levels (CALs) and gingival recession, and provides an accurate, quantified local inflammation burden (Nesse et al. 2008). Developing a tool for predicting systemic health outcomes by PISA could represent a key step in the prevention and management of multiple chronic conditions.

Periodontal assessment in non‐dental settings is a public health challenge. A previous population‐based study highlighted how several factors, including ethnicity, income level and education level, could predict the prevalence of periodontitis (Borrell et al. 2008). More objective evaluations including salivary biomarkers such as interleukin‐1β (IL‐1β) and matrix metalloproteinase‐8 (MMP‐8) have recently been explored as predictors of periodontal health and disease (Grant et al. 2022; Zhang et al. 2021).

Machine learning approaches offer key advantages when analysing high‐volume, high‐dimensional and multi‐modal data produced in biomedical research (Sajda 2006). Their application to dental datasets is in its infancy but holds great promise in view of the enhanced accuracy in identifying diagnostic patterns (Maity and Das 2017).

The aims of this study were to develop machine learning methods to (a) explore the association between periodontal inflammation and markers of systemic inflammation and metabolic health, and (b) preliminarily assess periodontal status based on systemic health indicators.

## 2 Methods

### 2.1 Study Design

This study was conducted in accordance with the Transparent Reporting of a multivariable prediction model for Individual Prognosis Or Diagnosis of artificial intelligence (TRIPOD + AI) guidelines (Collins et al. 2024). This study included the analyses of three datasets. A cross‐sectional sample of patients with periodontitis and healthy controls was first used to investigate the study aims. This was followed by validation analyses using two large US surveys: one that combined the 2001–2002 and 2003–2004 waves of the National Health and Nutrition Examination Survey (NHANES), and another one that used the 2009–2010 NHANES wave.

### 2.2 Local Dataset

Data were retrieved from a large cohort of participants recruited at the UCL Eastman Dental Institute and Hospitals between 2001 and 2018, as previously described in detail (Muñoz Aguilera et al. 2021). All participants provided written informed consent at the time of study participation, including the use of data for future analyses, and ethics approval was obtained from the local UCL Research Ethics Committee (Project ID: 16989/001).

#### 2.2.1 Inclusion Criteria

Cases were individuals ≥ 18 years old. All participants were systemically healthy, with no other systemic conditions, and had not received periodontal therapy within 6 months prior to the study assessment.

#### 2.2.2 Exclusion Criteria

Participants were excluded if they had any of the following: (1) active infectious diseases (e.g., hepatitis, HIV, or tuberculosis); (2) confirmed systemic diseases, including diabetes, kidney, liver, or cardiovascular diseases, hypertension, cancer, or were on chronic medication; (3) pregnancy or breastfeeding; or (4) regular use of non‐steroidal anti‐inflammatory drugs or antibiotics within 3 months prior to assessment.

#### 2.2.3 Periodontal Examination and Blood Sample Assessments

Periodontal assessment used a standardised protocol as previously described (D'Aiuto et al. 2006). PPD, BoP and margin alveolar bone loss were assessed. Case definition of periodontitis was confirmed against the latest validated classification (Tonetti et al. 2018). Fasting blood samples were collected at baseline and assessed for CRP (immunoturbidimetric assay, Cobas Integra, Roche Diagnostics, Mannheim, Germany; detection limit of 0.25 mg/L), while lipid fractions were quantified using standard clinical pathology procedures for analysis as previously described (D'Aiuto et al. 2006).

#### 2.2.4 Periodontal Inflamed Surface Area Calculation

PISA was calculated with the formula as previously described (Nesse et al. 2008). Both PPD and BoP were included in the calculation.

#### 2.2.5 Additional Variables

Socio‐demographics information was collected, including age, gender (male/female), ethnicity (Caucasian, Asian, Black African, Black Caribbean, other), body mass index (BMI, calculated as weight divided by the square of height) and smoking habits (smokers, non‐smokers and former smokers).

### 2.3 Validation Datasets

Data were retrieved from the 2001–2002, 2003–2004 and 2009–2010 NHANES datasets. NHANES is a cross‐sectional, population‐based data survey about the health and nutritional status of the non‐institutionalised civilian population in the United States. These surveys were administered by the National Center for Health Statistics (NCHS) within the Centers for Disease Control and Prevention (CDC) in the United States, with approval from the NCHS Institutional Review Board. Written informed consent was obtained from all participants.

#### 2.3.1 Validation Dataset 1

Data were retrieved from the 2001–2002 and 2003–2004 NHANES datasets. The exclusion criteria are addressed in Table S1. Periodontal examination included assessment of PPD, CAL and BoP at three sites per tooth (mid‐buccal, mesial and distal) in two randomly selected quadrants—one in the maxilla and one in the mandible.

The calculation of PISA was done using modified formulas, as previously described (Botelho et al. 2021). First, the average PPD was calculated for each individual tooth based on the three site records. periodontal epthelial surface area (PESA) was then determined using the mean PPD. PISA for each tooth was calculated by multiplying PESA by the proportion of the three sites around the tooth with BoP. Finally, the sum of PISA was calculated (Table S2).

#### 2.3.2 Validation Dataset 2

Data were retrieved from the 2009–2010 NHANES datasets. Participants aged 30 years and older were eligible for the periodontal evaluation if they had at least one tooth (excluding third molars) and did not meet any of the health exclusion criteria (Dye et al. 2014). Full‐mouth PPD and CAL data were collected by trained dental hygienists, but no BoP was recorded in this cycle. Periodontitis was defined using the AAP/CDC criteria for population‐based surveillance (Eke et al. 2012).

In both validation datasets, demographic information (age, gender, ethnicity), smoking habits, BMI, serum CRP levels and/or lipid fractions were included in the validation analyses.

### 2.4 Statistical Analyses

#### 2.4.1 Regression Analyses

First, fractional polynomial regression analyses were conducted to examine the nonlinear relationship between PISA and systemic biomarkers (CRP, high‐density lipoprotein [HDL], low‐density lipoprotein [LDL], total cholesterol [TC], triglycerides [TG]) (Royston and Altman 1994). Further, simple/multiple linear regression and logistic regression analyses were conducted to examine the associations (Hosmer Jr. et al. 2013; Montgomery et al. 2021). CRP, TC and TG values were log‐transformed (log) to achieve a normal distribution, while HDL and LDL were square root–transformed (sqrt) (Box and Cox 1964). The results are reported as coefficients or odds ratios (ORs) with their corresponding confidence intervals (95%) and *p*‐values.

**FIGURE 1:**
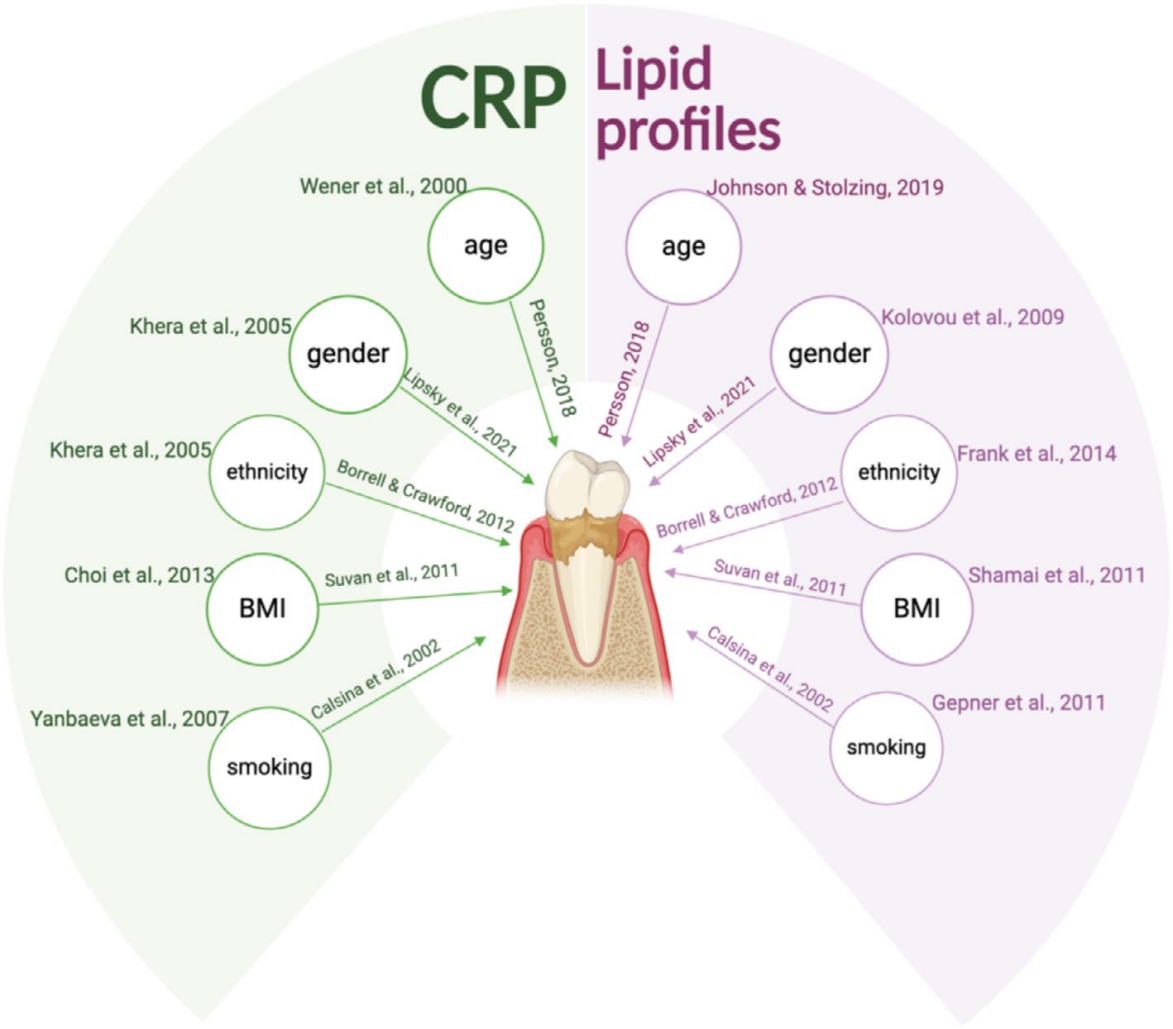
Evidence supporting the inclusion of the selected confounders. BMI, body mass index; CRP, C‐reactive protein.

Multiple models were adjusted for age (Johnson and Stolzing 2019; Persson 2018; Wener et al. 2000), gender (Khera et al. 2005; Kolovou et al. 2009; Lipsky et al. 2021), smoking (Calsina et al. 2002; Gepner et al. 2011; Yanbaeva et al. 2007), ethnicity (Borrell and Crawford 2012; Frank et al. 2014; Khera et al. 2005) and BMI (Choi et al. 2013; Shamai et al. 2011; Suvan et al. 2011), as these factors are recognised as a priori confounders according to previous evidence (VanderWeele 2019) (Figure 1).

Logistic regression was performed using a CRP level of 2 mg/L (Arnett et al. 2019; Fredriksson et al. 1999; Lee and Lee 2023), an LDL level of 3.3 mmol/L, a TC level of 5.2 mmol/L, a TG level of 2.2 mmol/L and an HDL level of either 1.0 mmol/L (male) or 1.3 mmol/L (female) as cut‐off points (Birtcher and Ballantyne 2004). Residual normality was assessed using a Q–Q plot for visual inspection (Wilk and Gnanadesikan 1968). Multicollinearity among independent variables was analysed using the variance inflation factor (VIF), with values above 10 indicating significant collinearity (Belsley et al. 1980). Analyses were conducted using the statistical software STATA MP (version 18.0, StataCorp LP, Texas, USA), setting the level of significance at 5%.

Random forest regressions (RFRs) were conducted for CRP, HDL, LDL, TC, TG and PISA scores for the feature importance Python (V3.12.1). RFR modelling of CRP against PISA was performed with a grid search for hyperparameter tuning (Bergstra and Bengio 2012) (Table S3).

#### 2.4.2 Prediction Approaches

Random forest (RF), support vector machine (SVM) and gradient boosting (GB) classifier models were employed for prediction. In these models, the synthetic minority over‐sampling technique (SMOTE) was applied to address class imbalance, and feature scaling was performed with StandardScaler. Hyperparameter tuning was conducted using grid search (Bergstra and Bengio 2012). To validate the models, 100 bootstrap samples were drawn from the training data, and the models were refitted on each sample to compute the area under the receiver operating characteristic curve (AUC). The final AUC was estimated as the average across all bootstrap samples. These analyses were conducted with ‘scikit‐learn’ library in Python (V3.12.1) (Pedregosa et al. 2011).

##### 2.4.2.1 From Local Inflammation Burden to Systemic Parameters

First, RF, SVM and GB used PISA, age, gender, ethnicity, smoking habits and BMI as inputs to predict the CRP classification. The optimal SVM model employed a radial basis function (RBF) kernel with *C* = 0.005 and *γ* = 0.01. The optimal RF model configuration included 100 estimators, a minimum samples split of 10, a minimum sample leaf of 1 and a maximum depth of 10. For the GB model, the optimal parameters were a learning rate of 0.2, a maximum depth of 5, a minimum samples leaf of 2, a minimum samples split of 5 and 100 estimators.

##### 2.4.2.2 From Systemic Parameters to Periodontal Status

Next, RF, SVM and GB were used, incorporating all systemic biomarkers (including CRP, HDL, LDL, TC, TG) along with the demographic variables (age, gender, ethnicity, BMI and smoking habits) to predict whether a participant had periodontitis or not. The optimal SVM model employed an RBF kernel with *C* = 0.1 and *γ* = 0.1. The optimal RF model configuration included 50 estimators, a minimum samples split of 5, a minimum samples leaf of 1 and a maximum depth of 20. The optimal GB model employed a learning rate of 0.4, a maximum depth of 7, a minimum samples leaf of 1, a minimum samples split of 8 and 200 estimators.

The performance of the classification model was evaluated using the receiver operating characteristic (ROC) curve and the AUC. The accuracy results derived from the AUC values were interpreted as low (0.50–0.70), moderate (0.71–0.90) or high (> 0.90) (Swets 1988).

## 3 Results

### 3.1 Study Participants

Data of 1061 participants from 10 clinical studies were retrieved as part of the first study analysis. After excluding studies lacking 6‐point PPD and/or BoP, serum CRP or lipid data, a final sample of 667 participants was obtained (Table 1). In the overall sample, 171 participants did not present with a diagnosis of periodontitis. PISA scores varied from 61 to 2481 mm^2^.

**TABLE 1:** Demographic information.

	Local dataset	Validation dataset 1 (2001–2002, 2003–2004 NHANES waves)	Validation dataset 2 (2009–2010 NHANES wave)
Number of participants	667	2288	664
Age, mean ± SD	43 ± 11	37 ± 14	54 ± 14
Gender			
Females	369 (55.32%)	1174 (51.31%)	275 (41.42%)
Males	298 (44.68%)	1114 (48.69%)	389 (58.58%)
Ethnicity	Caucasian 402 (60.27%)	Mexican American 550 (24.04%)	Mexican American 102 (15.36%)
	Asian 116 (17.39%)	Other Hispanic 71 (3.10%)	Other Hispanic 72 (10.84%)
	Black African 65 (9.75%)	Non‐Hispanic White 1243 (54.33%)	Non‐Hispanic White 354 (53.31%)
	Black Caribbean 59 (8.85%)	Non‐Hispanic Black 326 (14.25%)	Non‐Hispanic Black 104 (15.66%)
	Others 25 (3.74%)	Others 98 (4.28%)	Others 32 (4.82%)
Smoking habit			
Current smokers	121 (18.14%)	434 (18.97%)	279 (42.02%)
Never smoking	337 (50.53%)	1389 (60.71%)	22 (3.31%)
Former smokers	209 (31.33%)	465 (20.32%)	363 (54.67%)
Body mass index	26.29 ± 4.58	27.84 ± 6.12	28.94 ± 6.00

The validation dataset 1 comprised a total of 21,161 participants with demographic information retrieved from the NHANES datasets. Serum CRP levels were available for 15,610 participants, smoking habits were recorded for 5091 participants and BMI data were collected from 17,697 participants. Periodontal examinations were completed in 10,638 participants. After combining the available data, a final sample of 2288 participants was created for validation analysis (Tables 1 and S4). The PISA ranged from 0 to 2024 mm^2^.

The validation dataset 2 included a total of 10,537 participants with demographic information retrieved from the NHANES datasets. Serum CRP levels were available for 8299 participants, HDL and TC levels for 7846 participants, TG levels for 3357 participants and LDL levels for 3308 participants. Smoking habits were recorded for 2866 participants, and BMI data were available for 6730 participants. Periodontal examinations were completed in 4084 participants. After integrating the available data, a final sample of 664 participants was used for validation analysis (Tables 1 and S4).

### 3.2 Regression Analyses

First, fractional polynomial regression confirmed a nonlinear positive relationship between PISA and CRP values (optimal power of −1, *p* = 0.008), (Figure S1A). Furthermore, simple linear regression analysis confirmed an association between PISA and log(CRP); multiple linear regression analysis confirmed the same association (Table 2). No evidence of collinearity in the model was found (mean VIF = 1.11), and the assumptions were met (Figure S1B). The adjusted logistic regression analysis indicated that PISA scores and the likelihood of CRP ≥ 2 mg/L were positively associated (Table 3). Feature importance analysis showed that BMI (51.4%) had the highest importance in relation to CRP, followed by PISA (26.7%), with the remaining variables accounting for 21.9% (Figure S1C).

**TABLE 2:** Relationship between periodontal inflamed surface area (PISA) (cm^2^) and serum biomarkers.

Biomarkers	Coefficient	Standard error	*p*	95% CI
Log (CRP)				
Crude	0.0278	0.0057345	< 0.0001**	0.0165409 to 0.0390609
Adjusted	0.0199397	0.005691	< 0.0001**	0.0087651 to 0.0311144
Sqrt (HDL)				
Crude	−0.0114345	0.0084496	0.176	−0.0280267 to 0.0051578
Adjusted	−0.0048284	0.0090114	0.592	−0.0225243 to 0.0128676
Sqrt (LDL)				
Crude	0.0245079	0.0118459	0.039*	0.0012464 to 0.0477693
Adjusted	0.0162615	0.0127363	0.202	−0.008749 to 0.0412721
Log (TG)				
Crude	0.1275244	0.0352073	0.001**	0.0583889 to 0.1966599
Adjusted	0.0414838	0.0359358	0.249	−0.0290842 to 0.1120519
Log (TC)				
Crude	0.0070431	0.0089594	0.432	−0.0105502 to 0.0246365
Adjusted	0.0074309	0.0096851	0.443	−0.011588 to 0.0264499

*Note*: Log(CRP), log‐transformed C‐reactive protein; Sqrt (HDL), square root–transformed high‐density lipoprotein; Sqrt (LDL), square root–transformed low‐density lipoprotein; Log(TG): log‐transformed triglycerides; Log(TC), log‐transformed total cholesterol. *p ≤ 0.05, **p ≤ 0.01.

**TABLE 3:** Logistic regression analysis of biomarkers and periodontal inflamed surface area (PISA) (cm^2^).

Biomarkers		Odds ratio	95% CI	*p*‐value
C‐reactive protein				
Crude	< 2 mg/l ≥ 2 mg/l	1 (Ref) 1.048302	1.02378, 1.073412	< 0.0001**
Adjusted	< 2 mg/l ≥ 2 mg/l	1 (Ref) 1.036112	1.010618, 1.062249	0.005**
Low density lipid cholesterol				
Crude	≤ 3.3 mmol/l > 3.3 mmol/l	1 (Ref) 1.024024	1.002885, 1.045608	0.026*
Adjusted	≤ 3.3 mmol/l > 3.3 mmol/l	1 (Ref) 1.023656	1.00149, 1.046312	0.036*
Triglycerides				
Crude	≤ 2.2 mmol/l > 2.2 mmol/l	1 (Ref) 1.032984	1.010467, 1.056003	0.004**
Adjusted	≤ 2.2 mmol/l > 2.2 mmol/l	1 (Ref) 1.010834	0.9860611, 1.036228	0.395
High density lipid cholesterol				
Crude	< 1.0 mmol/l (male) ≥ 1.0 mmol/l (male)	1 (Ref) 1.029651	0.9714536, 1.091335	0.325
	< 1.3 mmol/l (female) ≥ 1.3 mmol/l (female)	1 (Ref) 0.9664963	0.9397995, 0.9939516	0.017*
Adjusted	< 1.0 mmol/l (male) ≥ 1.0 mmol/l (male)	1 (Ref) 1.030556	0.9664183, 1.098949	0.359
	< 1.3 mmol/l (female) ≥ 1.3 mmol/l (female)	1 (Ref) 0.9754804	0.9471238, 1.004686	0.099
Total cholesterol				
Crude	≤ 5.2 mmol/l	1 (Ref)		
	> 5.2 mmol/l	1.020526	1.00003, 1.041443	0.050*
Adjusted	≤ 5.2 mmol/l	1 (Ref)		
	> 5.2 mmol/l	1.019584	0.998106, 1.041425	0.074

*Note*: Adjusted models for C‐reactive protein, low‐density lipid cholesterol, triglycerides, total cholesterol were adjusted for body mass index, age, gender, ethnicity and smoking habit. Adjusted models for high‐density lipid cholesterol were adjusted for body mass index, age, ethnicity and smoking habit. *p ≤ 0.05, **p ≤ 0.01.

Simple linear regression analyses confirmed that PISA was only associated with sqrt(LDL) (*p* = 0.039) and log(TG) (*p* = 0.001) levels in the unadjusted models. Multiple linear regression analyses confirmed no substantial association between PISA and any of the lipid fractions (Table 2). Logistic regression analyses confirmed an association between PISA and LDL thresholds, independent of common confounders (Table 3). No association between PISA scores and HDL, LDL, TG and TC after polynomial regression models was observed (Table S5). Feature importance analysis confirmed that BMI consistently ranked as the most relevant variable for all lipid profiles. PISA was the second most important feature for HDL, LDL and TC, accounting for 18.4%, 21.3% and 32.3% of the feature importance, respectively (Figure S2).

### 3.3 Predication Approaches

**FIGURE 2:**
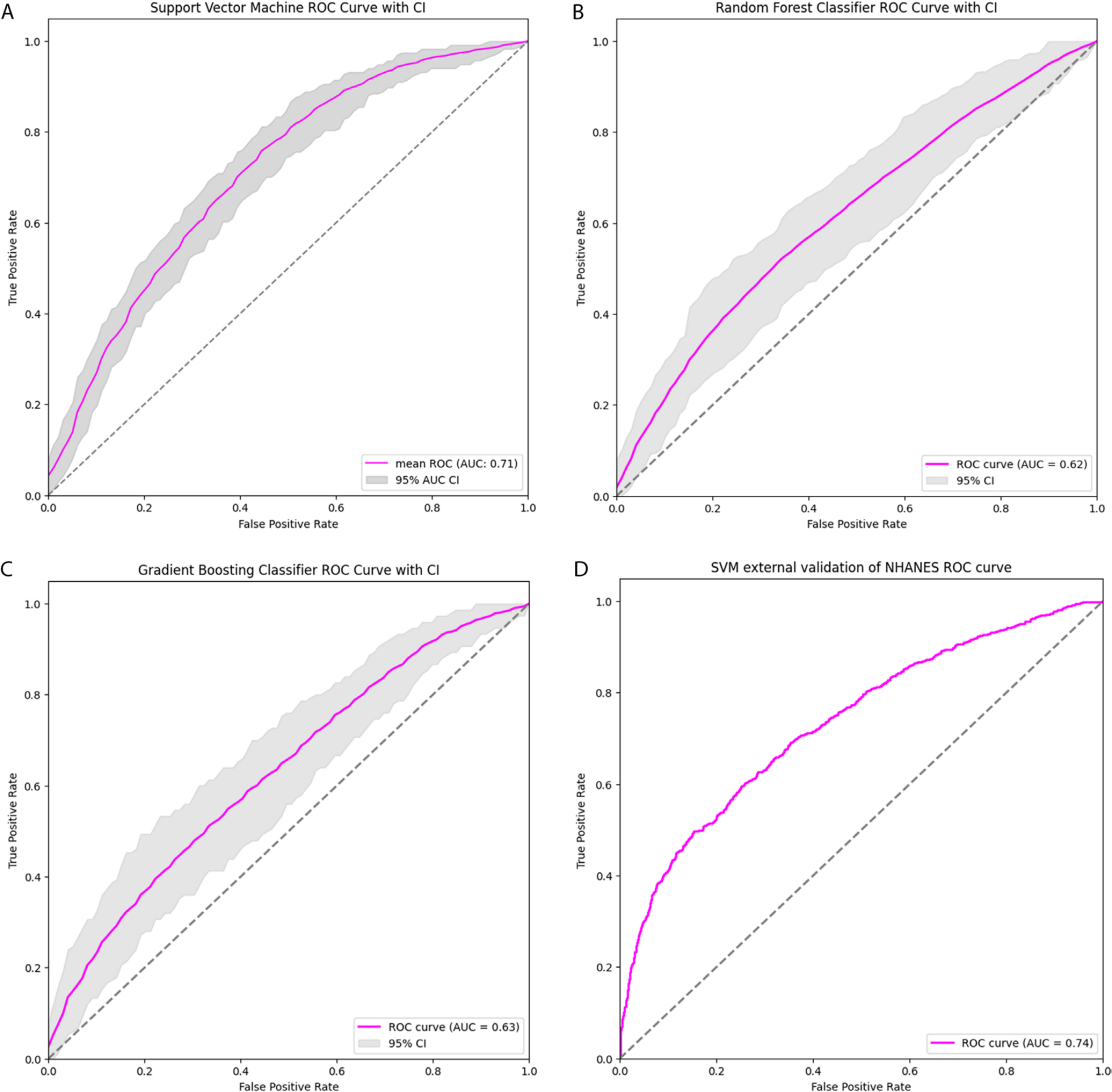
Receiver operating characteristic (ROC) curves and area under the curve (AUC) for the prediction of CRP thresholds (CRP < 2 and ≥ 2 mg/L): (A) Mean AUC = 0.71 across all bootstrap samples for the support vector machine (SVM) classifier. (B) Mean AUC = 0.62 across all bootstrap samples for the random forest (RF) classifier. (C) Mean AUC = 0.63 across all bootstrap samples for the gradient boosting (GB) classifier. (D) External validation yielding an AUC of 0.73.

Predictive analyses using CRP levels (thresholds of < 2 and ≥ 2 mg/L) against PISA scores resulted in three classification models—RF, SVM and GB. Among these models, SVM showed the highest discriminatory performance. The average AUC value across all bootstrap samples was 0.71 for the optimal model of SVM (Figure 2A). For the optimal model of RF, the mean AUC across bootstrap samples was 0.62 (Figure 2B). Lastly, the GB model resulted in an average AUC of 0.63 (Figure 2C). In the external validation, the best performing model (SVM) was applied to the validation dataset 1 (2001–2002 and 2003–2004 NHANES datasets) and showed an AUC of 0.74, indicating moderate discriminatory ability (Figure 2D) (sensitivity and specificity analyses are shown in Figure S3A,B).

**FIGURE 3:**
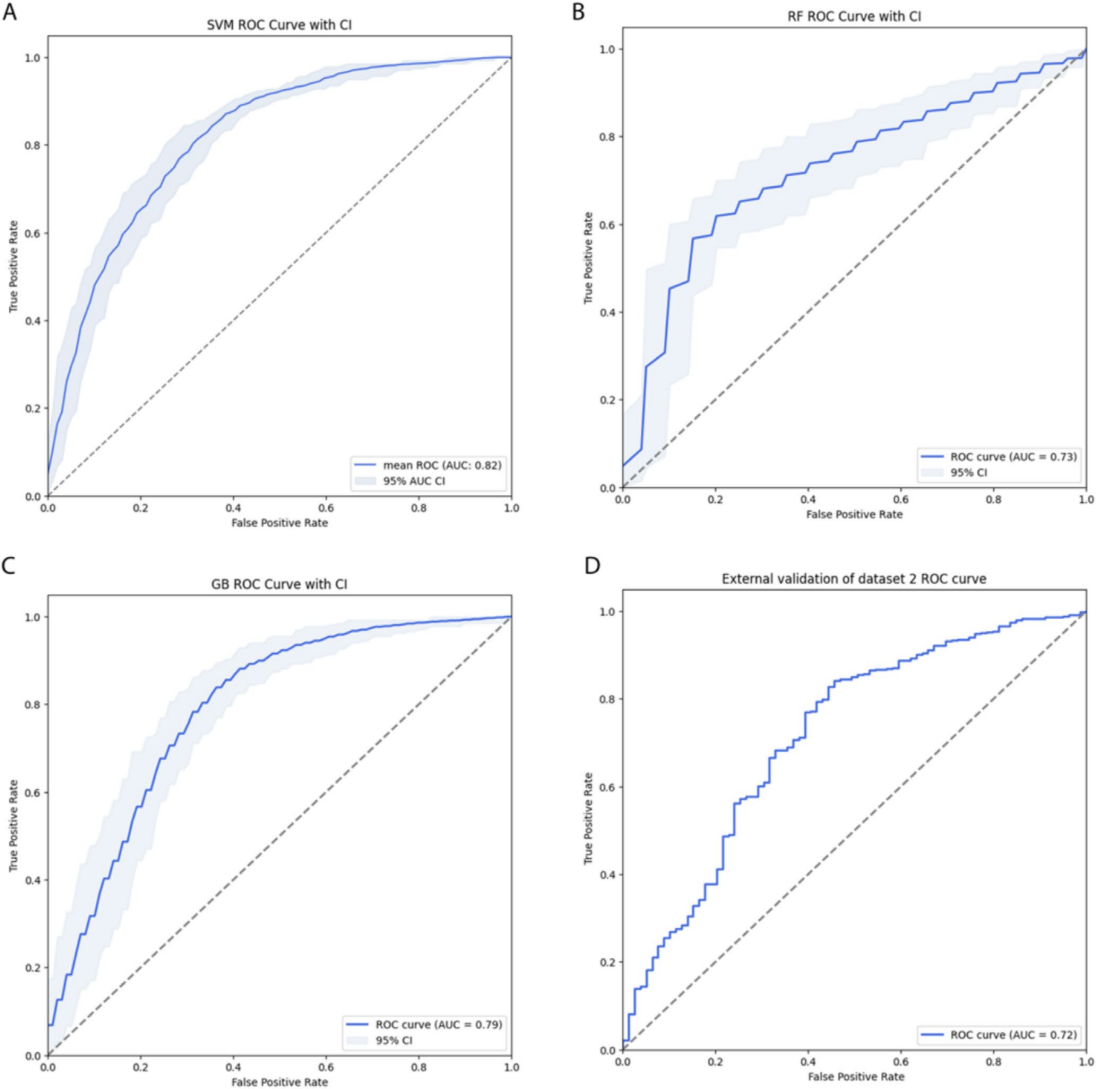
Receiver operating characteristic (ROC) curves and area under the curve (AUC) for the prediction of periodontal status (periodontitis vs. non‐periodontitis): (A) Mean AUC = 0.82 across all bootstrap samples for the support vector machine (SVM) classifier. (B) Mean AUC = 0.73 across all bootstrap samples for the random forest (RF) classifier. (C) Mean AUC = 0.79 across all bootstrap samples for the gradient boosting (GB) classifier. (D) External validation yielding an AUC of 0.72.

When assessing the prediction of periodontal status (periodontitis vs. non‐periodontitis) based on systemic indicators, the SVM model showed the best performance, achieving a mean AUC of 0.82 (Figure 3A). The optimal RF model showed a mean AUC of 0.73 (Figure 3B), while the GB model achieved a mean AUC of 0.79 (Figure 3C). In the external validation using validation dataset 2 (2009–2010 NHANES dataset), the SVM model was evaluated, showing an AUC of 0.72. This result indicates moderate discriminatory ability, suggesting that the model can distinguish between participants with and without periodontitis based on systemic indicators, albeit with a moderate level of accuracy (Figure 3D) (sensitivity and specificity at different thresholds are showed in Figure S3C,D).

## 4 Discussion

This study confirmed an association between the cumulative periodontal inflammatory burden (as assessed by PISA) and systemic inflammation (as assessed by serum CRP levels) in two separate datasets. Machine learning analyses indicated moderate discriminative abilities when periodontal inflammation was used to predict systemic inflammation (CRP ≥ 2 mg/L, AUCs of 0.72 and 0.74) and when using systemic health indicators to predict the diagnosis of periodontitis (AUCs of 0.82 and 0.72).

Previous studies have shown a relationship between PISA and CRP, particularly in older women and patients with end‐stage renal disease (Schöffer et al. 2021; Yoshihara et al. 2016), which aligns with the findings of our analysis. Based on fractional regression analysis, a PISA value of 1.5 cm^2^ could serve as a threshold for systemic effects of periodontitis. Interestingly, although PISA was associated with LDL levels, no independent association was observed between PISA and other lipid fractions (such as TG, HDL and TC). HDL levels were associated with PISA but only in females. This gender‐specific association has been previously reported (Mikami et al. 2021) and it may be attributed to gender‐related differences in lipid metabolism and inflammatory responses (Pascot et al. 2002). Although the adjusted model did not show direct associations between TG, HDL, TC and PISA, it is important to consider the possible mediating role of adiposity.

This study provides a novel approach to assessing the predictive ability of PISA on the extent of systemic inflammation (as assessed by CRP thresholds). CRP levels of 2 mg/L or greater were indeed linked to higher values of PISA. This low threshold of systemic inflammation had been previously linked to a diagnosis of periodontitis (Fredriksson et al. 1999; Paraskevas et al. 2008) as well as a validated risk‐enhancing factor to guide clinician–patient discussions on cardiovascular risk (Arnett et al. 2019). Prediction models could prove useful for exploring novel associations, refining existing theories and identifying high‐risk individuals for early intervention (Shmueli and Koppius 2011). Previous evidence had suggested using measures of gingival inflammation (full‐mouth bleeding score) as a predictor for systemic inflammation and subclinical atherosclerosis (Cairo et al. 2009). Further, PISA could have superior predictive capability for glycaemic levels compared to PPD, CAL and BoP (Susanto et al. 2012). The prediction of systemic host inflammation using cumulative measures of periodontitis had never been investigated before with multiple machine learning approaches. Despite periodontitis having been consistently associated with a number of chronic conditions such as cardiovascular diseases, diabetes and metabolic syndrome (Khumaedi et al. 2019), CRP, lipids and glycaemic levels are not routinely assessed in dental practice because of logistic and financial constraints. In this study, a machine learning classifier (SVM) incorporating PISA, BMI, age, gender, ethnicity and smoking status successfully predicted CRP thresholds. These levels, however, are influenced by additional factors, including genetic predisposition and lifestyle behaviour, which might explain the moderate AUC values (Fredrikson et al. 2004; Miller et al. 2005).

With the development of machine learning, saliva biomarkers have been explored for the prediction of periodontitis (Grant et al. 2022; Kim et al. 2021). Salivary biomarkers are, however, not routinely tested in general clinical practice and still pose some logistic challenges. In this study, instead, periodontal status was predicted using common systemic indicators, offering new insights into how markers such as CRP and other systemic health indicators may support the early diagnosis of periodontitis in non‐dental settings. This adds to the value of screening using self‐reported measures of periodontitis (Deng et al. 2024). In this study, self‐reported periodontal data was not available; however, machine learning models using systemic health indicators (CRP, LDL, HDL, TG, TC), age, gender, ethnicity, smoking habit and BMI predicted periodontal status with reasonable accuracy. Future observational studies validating cost‐effective and predictive models at scale could help bridge the gap between medical and dental practitioners.

**FIGURE 4:**
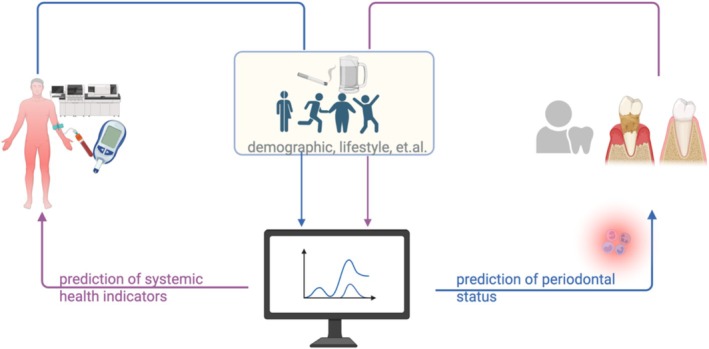
Two‐way predictive framework between systemic diseases and periodontitis.

Indeed, a predictive model such as the one proposed in this analysis provides a potential framework for integrating periodontal diseases into broader systemic health screening, facilitating early detection and risk stratification for chronic systemic conditions (such as diabetes and cardiovascular diseases) and periodontitis (Figure 4). The results of this study, however, must be interpreted with caution due to some limitations. First, not all potential confounders influencing CRP could be included, which likely contributed to the moderate AUC values observed in the predictive model. Second, in the external validation dataset 1 (NHANES 2001–2002 and 2003–2004), periodontal parameters were assessed in only two randomly selected quadrants and three sites per tooth, potentially underestimating the severity of periodontitis (Eke et al. 2010). In the external validation dataset 2 (NHANES 2009–2010), periodontitis was defined according to the CDC/AAP criteria (Eke et al. 2012), whereas the training dataset followed the 2017 World Workshop on the Classification of Periodontal and Peri‐Implant Diseases (Tonetti et al. 2018). This discrepancy in the definitions of periodontitis may account for the reduced AUC observed in the external validation. Additionally, ethnicity classification differed between the NHANES and the primary dataset, which may have introduced misclassification bias. Another limitation of this study is the cross‐sectional nature of the datasets, which limits the ability to assess the causal directionality of the observed relationships between periodontitis/PISA and systemic health indicators. Nonetheless, this study employed multiple statistical approaches to explore the association, ensuring robust findings. The use of both internal and external validation further strengthens the reliability of our predictive models.

## 5 Conclusions

This study confirmed a nonlinear association between PISA and CRP levels while minimal association with LDL among all traditional lipid fractions. Cumulative periodontal inflammation could serve as a predictor of systemic host response via machine learning modelling. Conversely, diagnosis of periodontitis was predicted using modelling of the common biomarkers of health (CRP, LDL, HDL, TG, TC) as well as demographic (age, gender, ethnicity) and lifestyle variables (smoking habits, BMI). These analyses hold great potential when used in primary care settings and health screening programmes for early detection and systemic impact of periodontitis, especially when addressing the burden of common chronic conditions such as diabetes and cardiovascular diseases.

## Author Contributions

Y.Y. and F.D. contributed substantially to the conception and design of the study as well as the initial idea for this article. Y.Y. and J.S. were involved in the data organisation. Y.Y., P.S. and F.D. were involved in the analysis and interpretation of the data. Y.Y. and F.D. drafted the manuscript. All the authors revised the manuscript critically and gave approval to the final version to be published.

## Disclosure

The authors have nothing to report.

## Consent

All participants provided written informed consent at the time of study participation, including use of data for future analyses.

## Conflicts of Interest

The authors declare no conflicts of interest.

## Supporting information


**Data S1.** Supporting Information.

**Data S2.** Supporting Information.

## Data Availability

The data that support the findings of this study are available from the corresponding author upon reasonable request.
