# Supplementary material for: The Association of Periodontal Inflammation and Systemic Health Indicators: A Machine Learning Approach
Source: J Clin Periodontol. 2025 Jul 23;52(10):1466–77. doi: 10.1111/jcpe.70000 (PMC12420081; doi:10.1111/jcpe.70000)
Supplement: Supplementary file 1 — Data S1. Supporting Information. [file JCPE-52-1466-s002.docx]

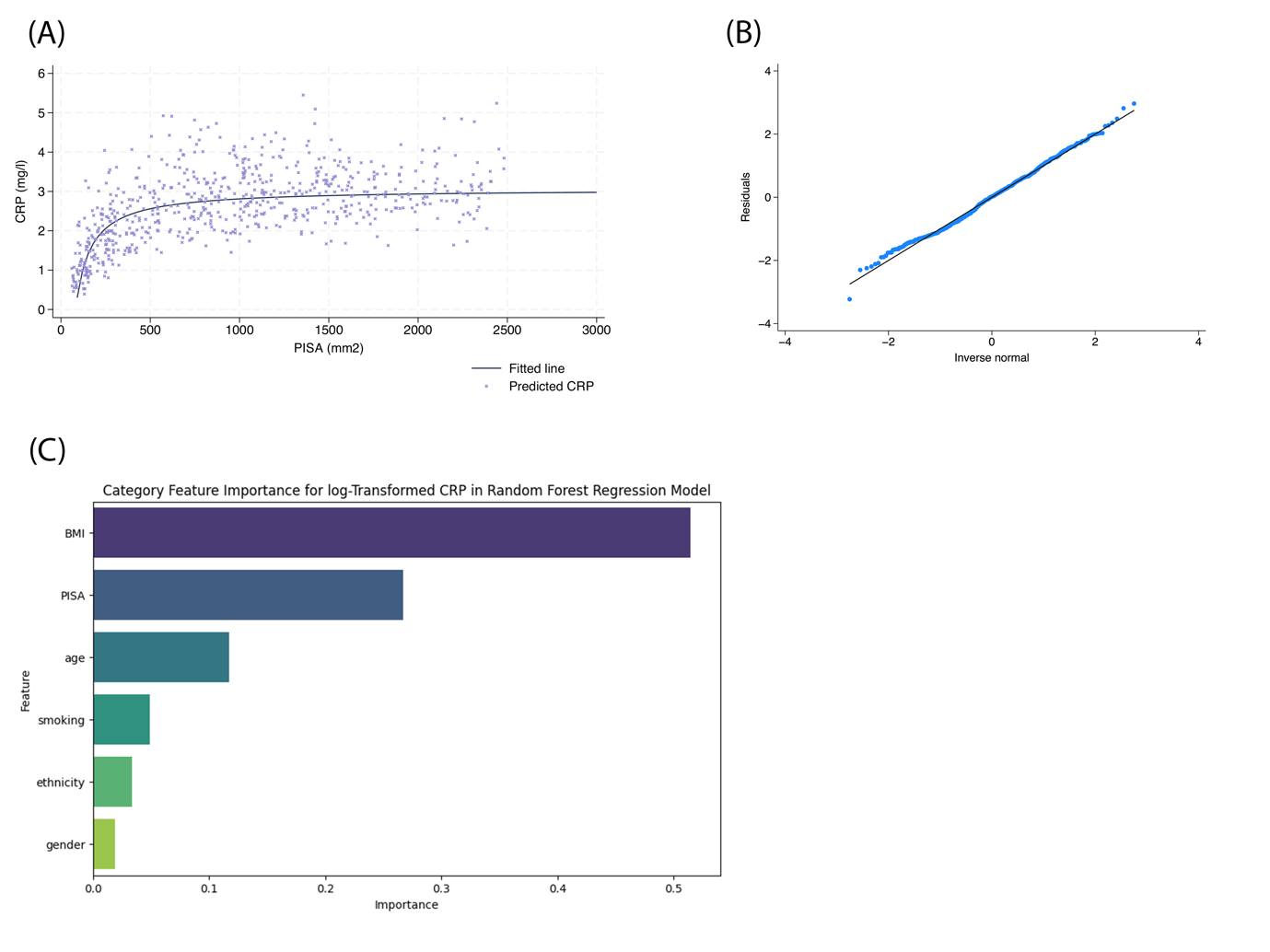


FIGURE S1. (A). Non-linear relationship between periodontal inflamed surface area (PISA) in mm^2^ and C-reactive protein (CRP) (mg/l); (B): Normal Q-Q Plot of Residuals for periodontal inflamed surface area and log-transformed C-reactive protein (CRP); (C). Feature importance of body mass index (BMI), PISA, age, gender, smoking and ethnicity on log-transformed CRP.


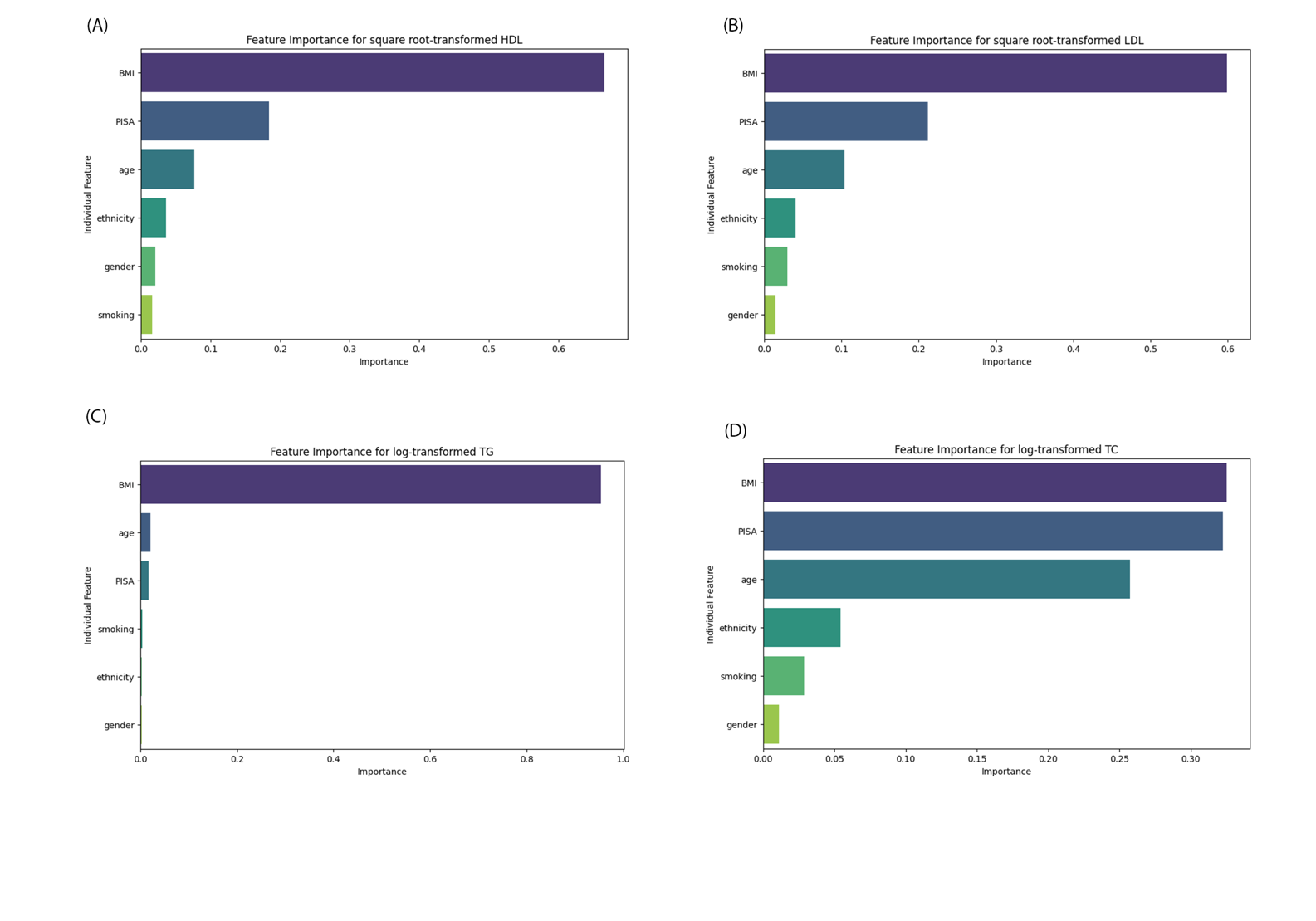


Figure S2. Feature importance of body mass index (BMI), PISA, age, gender, smoking and ethnicity on (A). sqrt-transformed high-density lipoprotein (HDL), (B). sqrt-transformed low-density lipoprotein LDL, (C).log-transformed triglycerides (TG), (D). log-transformed total cholesterol (TC).


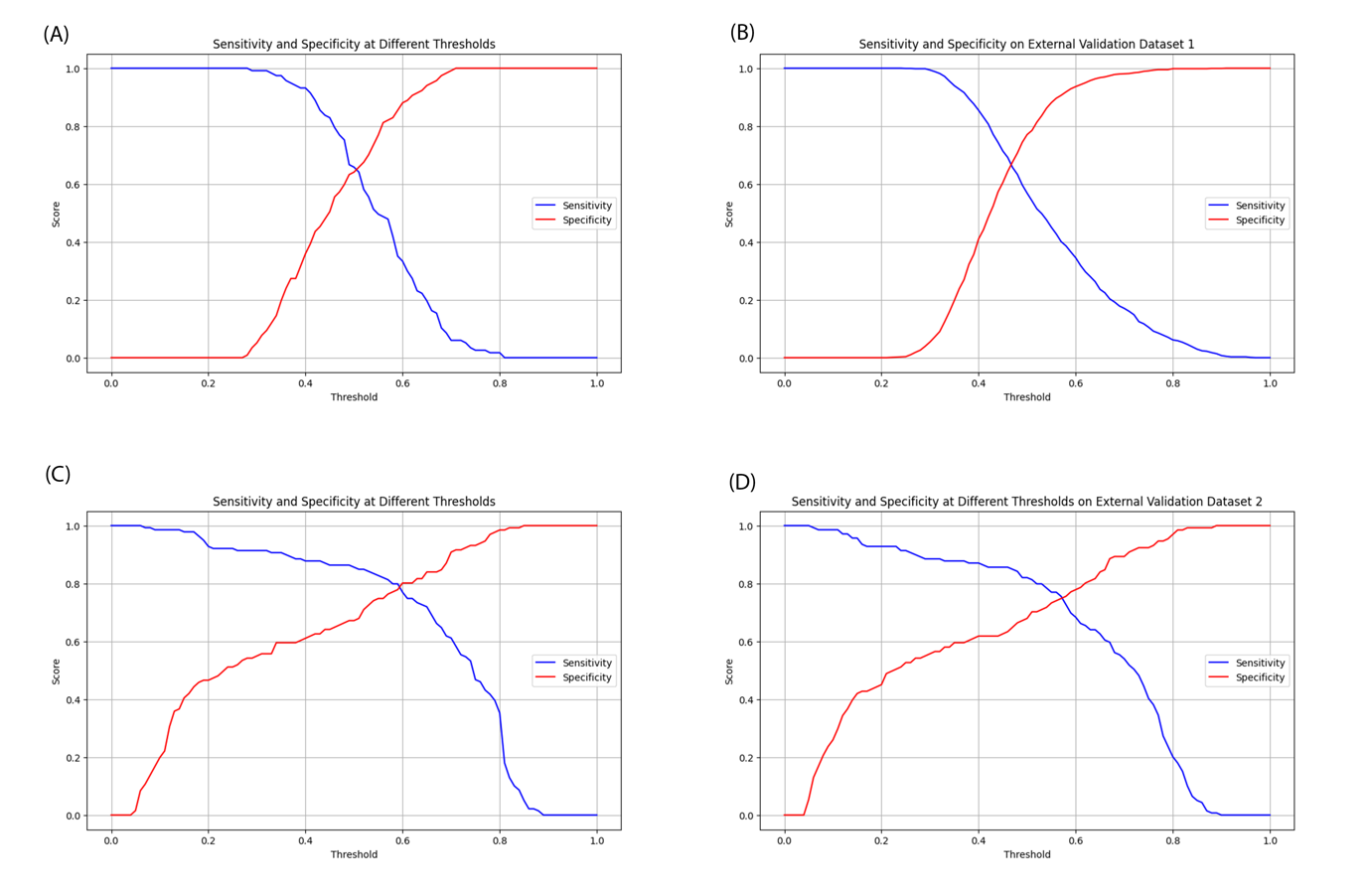


Figure S3. (A, B). The specificity and sensitivity in the development and external validation cohort for the prediction from local inflammation burden to systemic parameters, (C, D). The specificity and sensitivity in the development and external validation cohort for the prediction from systemic parameters to periodontal status
