# Supplementary material for: The Association of Periodontal Inflammation and Systemic Health Indicators: A Machine Learning Approach
Source: J Clin Periodontol. 2025 Jul 23;52(10):1466–77. doi: 10.1111/jcpe.70000 (PMC12420081; doi:10.1111/jcpe.70000)
Supplement: Supplementary file 2 — Data S2. Supporting Information. [file JCPE-52-1466-s001.docx]

Table S1: Exclusion criteria for 2001-2002, 2003-2004 National Health and Nutrition Examination Surveys (NHANES)

| Exclusion criteria for 2001-2002, 2003-2004 NHANES | Participants were excluded on the basis of the following criteria: including congenital heart murmurs, heart valve problems, congenital heart disease, bacterial endocarditis, rheumatic fever, kidney disease requiring dialysis, haemophilia, pacemaker or automatic defibrillator, or artificial material in heart veins or arteries requiring prophylactic antibiotics before dental examinations. |
| --- | --- |

Table S2: Calculation of Periodontal Inflamed Surface Area (PISA)

| **Tooth site** | **Formulas** |
| --- | --- |
| 17 | PESA17 =((25.4265*(((db17+b17+mb17)/3)^1))+(4.6241*(((db17+b17+mb17)/3)^2))+(-3.0787*(((db17+b17+mb17)/3)^3))+(0.95774*(((db17+b17+mb17)/3)^4))+(-0.10923*(((db17+b17+mb17)/3)^5))+(0.0040876*(((db17+b17+mb17)/3)^6))) |
| 16 | PESA16 =(16.8835*(((db16+b16+mb16)/3)^1))+(-0.5688*(((db16+b16+mb16)/3)^2))+(1.5433*(((db16+b16+mb16)/3)^3))+(-0.06519*(((db16+b16+mb16)/3)^4))+(-0.01454*(((db16+b16+mb16)/3)^5))+(0.0009019*(((db16+b16+mb16)/3)^6)) |
| 15 | PESA15=(39.2681*(((db15+b15+mb15)/3)^1))+(-7.3113*(((db15+b15+mb15)/3)^2))+(1.234*(((db15+b15+mb15)/3)^3))+(-0.12192*(((db15+b15+mb15)/3)^4))+(0.00626*(((db15+b15+mb15)/3)^5))+(-0.000126*(((db15+b15+mb15)/3)^6)) |
| 14 | PESA14=(21.8618*(((db14+b14+mb14)/3)^1))+(-2.3031*(((db14+b14+mb14)/3)^2))+(0.533*(((db14+b14+mb14)/3)^3))+(-0.04075*(((db14+b14+mb14)/3)^4))+(0.00062*(((db14+b14+mb14)/3)^5))+(0.0000119*(((db14+b14+mb14)/3)^6)) |
| 13 | PESA13=(16.5369*(((db13+b13+mb13)/3)^1))+(1.601*(((db13+b13+mb13)/3)^2))+(-0.2494*(((db13+b13+mb13)/3)^3))+(0.01087*(((db13+b13+mb13)/3)^4))+(0.00021*(((db13+b13+mb13)/3)^5))+(-0.0000182*(((db13+b13+mb13)/3)^6)) |
| 12 | PESA12=(18.7571*(((db12+b12+mb12)/3)^1))+(-1.6471*(((db12+b12+mb12)/3)^2))+(0.5258*(((db12+b12+mb12)/3)^3))+(-0.079*(((db12+b12+mb12)/3)^4))+(0.00589*(((db12+b12+mb12)/3)^5))+(-0.0001855*(((db12+b12+mb12)/3)^6)) |
| 11 | PESA11=(12.3905*(((db11+b11+mb11)/3)^1))+(0.1374*(((db11+b11+mb11)/3)^2))+(0.6717*(((db11+b11+mb11)/3)^3))+(-0.14536*(((db11+b11+mb11)/3)^4))+(0.01126*(((db11+b11+mb11)/3)^5))+(-0.0003083*(((db11+b11+mb11)/3)^6)) |
| 21 | PESA21=(12.3905*(((db21+b21+mb21)/3)^1))+(0.1374*(((db21+b21+mb21)/3)^2))+(0.6717*(((db21+b21+mb21)/3)^3))+(-0.14536*(((db21+b21+mb21)/3)^4))+(0.01126*(((db21+b21+mb21)/3)^5))+(-0.0003083*(((db21+b21+mb21)/3)^6)) |
| 22 | PESA22=(18.7571*(((db22+b22+mb22)/3)^1))+(-1.6471*(((db22+b22+mb22)/3)^2))+(0.5258*(((db22+b22+mb22)/3)^3))+(-0.079*(((db22+b22+mb22)/3)^4))+(0.00589*(((db22+b22+mb22)/3)^5))+(-0.0001855*(((db22+b22+mb22)/3)^6)) |
| 23 | PESA23=(16.5369*(((db23+b23+mb23)/3)^1))+(1.601*(((db23+b23+mb23)/3)^2))+(-0.2494*(((db23+b23+mb23)/3)^3))+(0.01087*(((db23+b23+mb23)/3)^4))+(0.00021*(((db23+b23+mb23)/3)^5))+(-0.0000182*(((db23+b23+mb23)/3)^6)) |
| 24 | PESA24=(21.8618*(((db24+b24+mb24)/3)^1))+(-2.3031*(((db24+b24+mb24)/3)^2))+(0.533*(((db24+b24+mb24)/3)^3))+(-0.04075*(((db24+b24+mb24)/3)^4))+(0.00062*(((db24+b24+mb24)/3)^5))+(0.0000119*(((db24+b24+mb24)/3)^6)) |
| 25 | PESA25=(39.2681*(((db25+b25+mb25)/3)^1))+(-7.3113*(((db25+b25+mb25)/3)^2))+(1.234*(((db25+b25+mb25)/3)^3))+(-0.12192*(((db25+b25+mb25)/3)^4))+(0.00626*(((db25+b25+mb25)/3)^5))+(-0.000126*(((db25+b25+mb25)/3)^6)) |
| 26 | PESA26=(16.8835*(((db26+b26+mb26)/3)^1))+(-0.5688*(((db26+b26+mb26)/3)^2))+(1.5433*(((db26+b26+mb26)/3)^3))+(-0.06519*(((db26+b26+mb26)/3)^4))+(-0.01454*(((db26+b26+mb26)/3)^5))+(0.0009019*(((db26+b26+mb26)/3)^6)) |
| 27 | PESA27=(25.4265*(((db27+b27+mb27)/3)^1))+(4.6241*(((db27+b27+mb27)/3)^2))+(-3.0787*(((db27+b27+mb27)/3)^3))+(0.95774*(((db27+b27+mb27)/3)^4))+(-0.10923*(((db27+b27+mb27)/3)^5))+(0.0040876*(((db27+b27+mb27)/3)^6)) |
| 47 | PESA47=(46.6148*(((db47+b47+mb47)/3)^1))+(-43.1558*(((db47+b47+mb47)/3)^2))+(16.75778*(((db47+b47+mb47)/3)^3))+(-2.48858*(((db47+b47+mb47)/3)^4))+(0.16174*(((db47+b47+mb47)/3)^5))+(-0.0038873*(((db47+b47+mb47)/3)^6)) |
| 46 | PESA46=(19.1229*(((db46+b46+mb46)/3)^1))+(-12.2566*(((db46+b46+mb46)/3)^2))+(5.575*(((db46+b46+mb46)/3)^3))+(-0.78145*(((db46+b46+mb46)/3)^4))+(0.04566*(((db46+b46+mb46)/3)^5))+(-0.0009711*(((db46+b46+mb46)/3)^6)) |
| 45 | PESA45=(13.1705*(((db45+b45+mb45)/3)^1))+(5.0958*(((db45+b45+mb45)/3)^2))+(-1.0989*(((db45+b45+mb45)/3)^3))+(0.10864*(((db45+b45+mb45)/3)^4))+(-0.00559*(((db45+b45+mb45)/3)^5))+(0.0001179*(((db45+b45+mb45)/3)^6)) |
| 44 | PESA44=(24.6866*(((db44+b44+mb44)/3)^1))+(-4.8531*(((db44+b44+mb44)/3)^2))+(1.3992*(((db44+b44+mb44)/3)^3))+(-0.18028*(((db44+b44+mb44)/3)^4))+(0.01037*(((db44+b44+mb44)/3)^5))+(-0.0002229*(((db44+b44+mb44)/3)^6)) |
| 43 | PESA43=(24.6992*(((db43+b43+mb43)/3)^1))+(-3.5868*(((db43+b43+mb43)/3)^2))+(0.6903*(((db43+b43+mb43)/3)^3))+(-0.05799*(((db43+b43+mb43)/3)^4))+(0.00189*(((db43+b43+mb43)/3)^5))+(-0.0000142*(((db43+b43+mb43)/3)^6)) |
| 42 | PESA42=(16.4395*(((db42+b42+mb42)/3)^1))+(-1.0337*(((db42+b42+mb42)/3)^2))+(0.4146*(((db42+b42+mb42)/3)^3))+(-0.05711*(((db42+b42+mb42)/3)^4))+(0.00257*(((db42+b42+mb42)/3)^5))+(-0.0000211*(((db42+b42+mb42)/3)^6)) |
| 41 | PESA41=(21.46*(((db41+b41+mb41)/3)^1))+(-6.6888*(((db41+b41+mb41)/3)^2))+(2.4638*(((db41+b41+mb41)/3)^3))+(-0.39094*(((db41+b41+mb41)/3)^4))+(0.02743*(((db41+b41+mb41)/3)^5))+(-0.0007116*(((db41+b41+mb41)/3)^6)) |
| 31 | PESA31=(21.46*(((db31+b31+mb31)/3)^1))+(-6.6888*(((db31+b31+mb31)/3)^2))+(2.4638*(((db31+b31+mb31)/3)^3))+(-0.39094*(((db31+b31+mb31)/3)^4))+(0.02743*(((db31+b31+mb31)/3)^5))+(-0.0007116*(((db31+b31+mb31)/3)^6)) |
| 32 | PESA32=(16.4395*(((db32+b32+mb32)/3)^1))+(-1.0337*(((db32+b32+mb32)/3)^2))+(0.4146*(((db32+b32+mb32)/3)^3))+(-0.05711*(((db32+b32+mb32)/3)^4))+(0.00257*(((db32+b32+mb32)/3)^5))+(-0.0000211*(((db32+b32+mb32)/3)^6)) |
| 33 | PESA33=(24.6992*(((db33+b33+mb33)/3)^1))+(-3.5868*(((db33+b33+mb33)/3)^2))+(0.6903*(((db33+b33+mb33)/3)^3))+(-0.05799*(((db33+b33+mb33)/3)^4))+(0.00189*(((db33+b33+mb33)/3)^5))+(-0.0000142*(((db33+b33+mb33)/3)^6)) |
| 34 | PESA34=(24.6866*(((db34+b34+mb34)/3)^1))+(-4.8531*(((db34+b34+mb34)/3)^2))+(1.3992*(((db34+b34+mb34)/3)^3))+(-0.18028*(((db34+b34+mb34)/3)^4))+(0.01037*(((db34+b34+mb34)/3)^5))+(-0.0002229*(((db34+b34+mb34)/3)^6)) |
| 35 | PESA35=(13.1705*(((db35+b35+mb35)/3)^1))+(5.0958*(((db35+b35+mb35)/3)^2))+(-1.0989*(((db35+b35+mb35)/3)^3))+(0.10864*(((db35+b35+mb35)/3)^4))+(-0.00559*(((db35+b35+mb35)/3)^5))+(0.0001179*(((db35+b35+mb35)/3)^6)) |
| 36 | PESA36=(19.1229*(((db36+b36+mb36)/3)^1))+(-12.2566*(((db36+b36+mb36)/3)^2))+(5.575*(((db36+b36+mb36)/3)^3))+(-0.78145*(((db36+b36+mb36)/3)^4))+(0.04566*(((db36+b36+mb36)/3)^5))+(-0.0009711*(((db36+b36+mb36)/3)^6)) |
| 37 | PESA37=(46.6148*(((db37+b37+mb37)/3)^1))+(-43.1558*(((db37+b37+mb37)/3)^2))+(16.75778*(((db37+b37+mb37)/3)^3))+(-2.48858*(((db37+b37+mb37)/3)^4))+(0.16174*(((db37+b37+mb37)/3)^5))+(-0.0038873*(((db37+b37+mb37)/3)^6)) |
| 17 | PISA17= (bop17db + bop17b+ bop17mb)/3* PESA17 |
| 16 | PISA16= (bop16db+bop16b+bop16mb)/3* PESA16 |
| 15 | PISA15= (bop15db+bop15b+bop15mb)/3* PESA15 |
| 14 | PISA14= (bop14db+bop14b+bop14mb)/3* PESA14 |
| 13 | PISA13= (bop13db+bop13b+bop13mb)/3* PESA13 |
| 12 | PISA12= (bop12db+bop12b+bop12mb)/3* PESA12 |
| 11 | PISA11= (bop11db+bop11b+bop11mb)/3* PESA11 |
| 21 | PISA21= (bop21db+bop21b+bop21mb)/3* PESA21 |
| 22 | PISA22= (bop22db+bop22b+bop22mb)/3* PESA22 |
| 23 | PISA23= (bop23db+bop23b+bop23mb)/3* PESA23 |
| 24 | PISA24= (bop24db+bop24b+bop24mb)/3* PESA24 |
| 25 | PISA25= (bop25db+bop25b+bop25mb)/3* PESA25 |
| 26 | PISA26= (bop26db+bop26b+bop26mb)/3* PESA26 |
| 27 | PISA27= (bop27db+bop27b+bop27mb)/3* PESA27 |
| 47 | PISA47=(bop47db+bop47b+bop47mb)/3* PESA47 |
| 46 | PISA46=(bop46db+bop46b+bop46mb)/3* PESA46 |
| 45 | PISA45=(bop45db+bop45b+bop45mb)/3* PESA45 |
| 44 | PISA44=(bop44db+bop44b+bop44mb)/3* PESA44 |
| 43 | PISA43=(bop43db+bop43b+bop43mb)/3* PESA43 |
| 42 | PISA42=(bop42db+bop42b+bop42mb)/3* PESA42 |
| 41 | PISA41=(bop41db+bop41b+bop41mb)/3* PESA41 |
| 31 | PISA31=(bop31db+bop31b+bop31mb)/3* PESA31 |
| 32 | PISA32=(bop32db+bop32b+bop32mb)/3* PESA32 |
| 33 | PISA33=(bop33db+bop33b+bop33mb)/3* PESA33 |
| 34 | PISA34=(bop34db+bop34b+bop34mb)/3* PESA34 |
| 35 | PISA35=(bop35db+bop35b+bop35mb)/3* PESA35 |
| 36 | PISA36=(bop36db+bop36b+bop36mb)/3* PESA36 |
| 37 | PISA37=(bop37db+bop37b+bop37mb)/3* PESA37 |
| PISA half mouth | PISA half=(PISA11+PISA12+PISA13+PISA14+PISA15+PISA16+PISA17+PISA21+PISA22+PISA23+PISA24+PISA25+PISA26+PISA27+PISA31+PISA32+PISA33+PISA34+PISA35+PISA36+PISA37+PISA41+PISA42+PISA43+PISA44+PISA45+PISA46+PISA47) |
| PISA full mouth | PISA full month=PISA half*2 |

PISA and PESA were calculated using in the following steps: (1) Calculation of mean PPD each tooth, (2) Mean PPD is used to calculate PESA for each tooth,(3) For each tooth, PISA is calculated through the multiplication of PESA by the proportion of sites around the tooth with BOP, (4)The sum of all individual PISA and PESA scores, in mm2, (5) The data calculated in step (4) were multiplied by 2 to account for the fact that only two randomly selected quadrants were included for each participant. db: probing pocket depth (PPD) at distal-buccal, b: PPD at mid-buccal, mb: PPD at mesial-buccal, bop(tooth site)db: Bleeding on Probing (BoP) at distal-buccal, bop(tooth site)b: Bleeding on Probing (BoP) at mid-buccal, bop(tooth site)mb: Bleeding on Probing (BoP) at mesial-buccal.

Table S3: Optimal parameter of random forest regressions (RFR) for C-reactive Protein (CRP), total cholesterol (TC), triglycerides (TG), low density lipid protein (LDL), high density lipid protein (HDL)

| Biomarkers | Optimal Parameters |
| --- | --- |
| CRP | max depth of 10, min samples leaf of 16, min samples split of 5, and n_estimators of 400 |
| HDL | max depth = 10, min samples leaf = 32, min samples split = 2, and n_estimators = 200 |
| LDL | max depth = 5, min samples leaf = 24, min samples split = 5, and n_estimators = 500 |
| TG | max depth = 5, min samples leaf = 16, min samples split = 5, and n_estimators = 500 |
| TC | max depth = 5, min samples leaf = 4, min samples split = 15, and n_estimators = 100 |

Table S4: Amount of missing data in the validation datasets

| **Variables** | **Dataset 1 (N=21161)** | | **Dataset 2(N=10537)** | |
| --- | --- | --- | --- | --- |
|  | **Collected data, N (%)** | **Missing data, N (%)** | **Collected data, N (%)** | **Missing data, N (%)** |
| Age | 21161 (100.0) | 0(0.0) | 10537 (100.0) | 0(0.0) |
| gender | 21161 (100.0) | 0(0.0) | 10537 (100.0) | 0(0.0) |
| Ethnicity | 21161 (100.0) | 0(0.0) | 10537 (100.0) | 0(0.0) |
| BMI | 17697 (83.6) | 3464 (16.4) | 6730 (63.9) | 3807 (36.1) |
| Serum CRP | 15610 (73.8) | 5551 (26.2) | 8299 (78.8) | 2238 (21.2) |
| Smoking habits | 5091 (24.1) | 16070 (75.9) | 2866 (27.2) | 7671 (72.8) |
| Periodontal examination | 10638 (50.3) | 10523 (49.7) | 4084 (38.8) | 6453 (61.2) |
| TC | NI | NI | 7846 (74.5) | 2691 (25.5) |
| TG | NI | NI | 3357 (31.9) | 7180 (68.1) |
| LDL | NI | NI | 3308 (31.4) | 7729 (68.6) |
| HDL | NI | NI | 7846 (74.5) | 2691 (25.5) |
| Participants with all included variables | 2288 (10.8) | 18873 (89.2) | 664 (6.3) | 9873 (93.7) |

BMI: body mass index, CRP:C-reactive Protein, TC: total cholesterol, TG: triglycerides, LDL: low density lipid protein, HDL: high density lipid protein, PPD: pocket probing depth, BoP: bleeding on probing, NI: not include in the models

Table S5: Relationship between periodontal inflamed surface area (PISA) (mm^2^) and lipid profiles in the polynomial regression models

|  | **Optimal powers of independent variable** | **coefficient** | **robust standard error** | **p value** | **95% CI** |
| --- | --- | --- | --- | --- | --- |
| High density lipid cholesterol | -2 | -.0002005 | .0001756 | 0.254 | -.0005453, .0001444 |
|  | 3 | .0020474 | .0032462 | 0.528 | -.0043272, .0084221 |
| Low density lipid cholesterol | -0.5 | .2132021 | .1397632 | 0.128 | -.0612518, .487656 |
|  | 0 | .2224509 | .1212764 | 0.067 | -.0157003, .4606022 |
| Triglycerides | -0.5 | -.4561479 | .3919257 | 0.245 | 6.40e-11, 1.11e-10 |
|  | -0.5 | -.1234216 | .110779 | 0.266 | -.0890156, .2356909 |
| Total cholesterol | -0.5 | -.2935844 | .2602926 | 0.260 | -.8047228, .217554 |
|  | -0.5 | -.0740495 | .0744942 | 0.321 | -.2203343, .0722353 |
